# Specification and Causal Pathway Diagramming of Implementation Strategies to Support Development of Access‐Enhancing Injectable HIV Treatment Programmes at Eight Diverse Health Clinics Across the United States

**DOI:** 10.1002/jia2.70190

**Published:** 2026-10-08

**Authors:** Kathrine Meyers, Nadia Nguyen, Charlie Ferrusi, Jason Zucker, Sarit A. Golub, Bryan A. Kutner, Mamta Karani, Cody A. Chastain, Marilyn Blasingame, Moya Brown‐Lopez, Jennifer Burdge, Delivette Castor, Caroline Carnevale, Jenna Eldib, Elizabeth Furuya, Omolola Akolo, Kelly Bastien, Melissa Curry, Will Holt, Bijou R. Hunt, Shin Ly, Carlotta Starks, Fabriel Torres, Marlene Matosky

**Affiliations:** ^1^ Aaron Diamond AIDS Research Center Columbia University Irving Medical Center New York City New York USA; ^2^ Division of Infectious Diseases Department of Medicine Columbia University Irving Medical Center New York City New York USA; ^3^ Hunter College of the City University of New York New York City New York USA; ^4^ Montefiore Medical Center and Albert Einstein College of Medicine Bronx New York USA; ^5^ New York City Health Department Queens New York USA; ^6^ Division of Infectious Diseases Department of Medicine Vanderbilt University Medical Center Nashville Tennessee USA; ^7^ NewYork‐Prebysterian Hospital New York City New York USA; ^8^ Baltimore City Health Department Baltimore Maryland USA; ^9^ Sunshine Care Center Florida Department of Health Orlando Florida USA; ^10^ Abounding Prosperity, Inc. Dallas Texas USA; ^11^ Coastal Family Health Center Biloxi Mississippi USA; ^12^ Sinai Infectious Disease Center Chicago Illinois USA; ^13^ Positive Impact Health Center Decatur Georgia USA; ^14^ Jack Martin Fund Center Mount Sinai Institute for Advanced Medicine New York City New York USA; ^15^ San Antonio AIDS Foundation San Antonio Texas USA; ^16^ U.S. Department of Health and Human Services Health Resources and Services Administration Rockville Maryland USA

**Keywords:** HIV, implementation science, long‐acting injectables, treatment

## Abstract

**Introduction:**

Long‐acting injectable antiretroviral therapy (LAI ART) represents a transformative development in HIV care because it may mitigate longstanding barriers that contribute to gaps in health outcomes. Providing LAI ART to people not consistently on HIV treatment or lacking treatment options requires intentional programme planning to overcome implementation barriers, without which only well‐resourced patients benefit, worsening health outcome gaps. The ALAI UP Project delivered clinic‐level implementation strategies to support eight clinics across Ending the HIV Epidemic jurisdictions to implement injectable cabotegravir and rilpivirine (iCAB/RPV).

**Methods:**

We specified seven implementation strategies and assessed their impact on clinic achievement of three key programme outcomes: (a) access to information about iCAB/RPV for all patients regardless of clinical eligibility; (b) access to iCAB/RPV regardless of healthcare coverage; and (c) access to iCAB/RPV for those who can clinically benefit the most, including patients who are unable to achieve durable viral suppression on oral ART for reasons other than cabotegravir or rilpivirine resistance. Implementation strategies were delivered from March 2023 through February 2025, and results are reported at the end of year 2 of the 3‐year project. We developed causal pathway diagrams (CPDs) for each strategy, identifying determinants, preconditions, moderators, mechanisms and outcomes.

**Results:**

ALAI UP strategies supported clinic progress towards all three outcomes: six clinics had partially or fully achieved access to information about iCAB/RPV for all patients, seven clinics had partially or fully achieved access to iCAB/RPV for patients regardless of healthcare coverage, and six clinics had provided access to iCAB/RPV for those who could benefit the most clinically. By developing CPDs for each strategy, we explicated how strategies operated across different clinical contexts. Through this process, we identified mechanisms of action that needed to be activated, moderators that impacted strategy strength and preconditions that needed to be in place for the strategy to function. These insights informed changes to strategies in the final year of the project.

**Conclusion:**

This work demonstrates that applying implementation science tools to service delivery projects can enhance precision and reproducibility of strategies, identify intervention points for improving strategies when mechanisms are not activated, and clarify hypothesized causal pathways testable in future hybrid effectiveness‐implementation trials.

## 1 Introduction

Long‐acting injectable antiretroviral therapy (LAI ART) represents a transformative development in HIV care because it presents an opportunity to mitigate longstanding barriers to care that contribute to gaps in health outcomes. LAI ART could close that gap if intentionally delivered to people not consistently on HIV treatment or lacking treatment options. These populations typically experience delayed access to novel biomedical interventions, resulting in persistent gaps in adoption among racial/ethnic groups, rural‐urban residence and insurance types [1, 2, 3].

Despite interest in injectable cabotegravir/rilpivirine (iCAB/RPV), its real‐world adoption remains low [4, 5]. Sparse existing estimates suggest that since its Food and Drug Administration approval as a once‐every‐2‐month injection regimen in January 2022, only 1%–2% of people with HIV have been prescribed iCAB/RPV in the United States [5, 6, 7]. This limited uptake stems from implementation complexity, leading to few clinics offering iCAB/RPV, and slow programme growth at clinics that do offer it [8, 9]. iCAB/RPV must be administered by a trained medical professional and is, therefore, most often delivered in clinical settings; but LAIs require new workflows, enhanced coordination including with specialty pharmacies, and substantial paperwork to ensure medication affordability, procurement, proper storage and timely injection [10]. Addressing these hurdles is urgent to improve access to iCAB/RPV and ensure swift implementation of future longer‐interval LAI ARTs across more clinical settings [11].

To counteract gaps in access resulting from uneven rollout of iCAB/RPV, the “Accelerating Implementation of Multilevel Strategies to Advance Long‐Acting Injectables for Underserved Populations” (the ALAI UP Project) was funded as a service delivery project in 2022 by the Health Resources and Services Administration (HRSA) HIV/AIDS Bureau and the Minority HIV/AIDS Fund Initiative. The ALAI UP project is a 4‐year implementation project, with participating clinics receiving funding support for 3 years. Using implementation science and behaviour change frameworks, ALAI UP designed and delivered a package of implementation strategies to support eight clinics across the United States in introducing and scaling LAI ART programmes in ways that enhanced access to LAI ART [12]. The goal of the project was not to support clinics to initiate the most patients on LAI ART, but rather to build programmes that explicitly prioritize education about and access to LAI ART for underserved populations and, in the longer term, reduce gaps in HIV outcomes. The ALAI UP team designed and delivered a package of implementation strategies to support clinics in building access‐enhancing LAI ART programmes that provide (a) access to information about iCAB/RPV for all patients regardless of clinical eligibility; (b) access to iCAB/RPV regardless of healthcare coverage; and (c) access to iCAB/RPV for those who can benefit the most clinically (e.g. people who are not able to achieve durable viral suppression with oral medication for reasons other than resistance to cabotegravir or rilpivirine). We operationalize these as programme outcomes to monitor the impact of our strategies. The manuscript reports findings from the midpoint of ALAI UP, corresponding to the end of year 2 within a 3‐year implementation facilitation period.

The objective of this manuscript is to demonstrate how implementation science can be used in service delivery projects to elucidate implementation processes so that they can be theory‐informed, improved in real time, and further refined and packaged in future studies designed to evaluate effectiveness. Specifically, we highlight the use of causal pathway diagrams (CPDs), graphical tools designed to map the chain of events through which implementation strategies operate and achieve intended outcomes, and focus on mechanisms to move beyond simple descriptions of implementation towards an explanation of how and why implementation succeeded or did not succeed.

Although our findings are grounded in the experience of the United States health system, identified challenges and strategies have broader relevance for international settings introducing iCAB/RPV within diverse financing, supply chain and workforce contexts. These findings may inform the adaptation and scale‐up of iCAB/RPV beyond the United States and highlight key considerations for implementation across global delivery systems.

## 2 Methods

Columbia Irving Medical Center Institutional Review Board reviewed and approved this protocol (AAAU7784).

### 2.1 Study Setting and Participants

#### 2.1.1 Strategy Recipient

Table S1 describes the eight participating clinics. Selected clinics were required to assemble a clinic implementation team (CIT), which consisted of a project champion and additional clinic staff, typically including: an administrator, clinician, pharmacist, data specialist and/or person with HIV. In some cases, one individual filled several of these roles.

#### 2.1.2 Strategy Deliverer

Implementation strategies were delivered to clinics by the ALAI UP implementation facilitation team (IFT), a multidisciplinary group of implementation scientists, people living with HIV, technical assistance (TA) providers, clinical educators, clinicians, pharmacists, epidemiologists and data analysts. A dedicated TA provider was assigned to each clinic to serve as the primary point of contact and provide individualized, hands‐on implementation coaching throughout the project. In addition, the dedicated TA provider brought in appropriate subject matter experts from the IFT as needed, for example, clinicians for Clinical TA or pharmacists for cost and coverage TA.

### 2.2 Implementation Strategies

We describe seven discrete implementation strategies the IFT delivered during the first 2 years of the project, from 1 March 2023, through 28 February 2025. Discrete strategies included: (1) a library of modifiable resources called the Resource Incubator (RI); (2) Implementation Coaching Technical Assistance (TA); (3) Clinical TA; (4) Cost and Coverage TA; (5) Motivational Interviewing Training; (6) In‐Person Multisite Convening; and (7) Virtual Communities of Practice (CoP). The strategies operated together as a multifaceted strategy [13] to move clinics towards the goal of having an access‐enhancing LAI ART programme. Strategy details are specified in Table S2 [14]. Clinics were provided USD ∼90,000 each year for 3 years.

### 2.3 Programme Outcomes

We posited that access‐enhancing LAI ART programmes are ones that provide (a) access to information about iCAB/RPV for all patients regardless of clinical eligibility; (b) access to iCAB/RPV regardless of insurance coverage; and (c) access to iCAB/RPV for those who can benefit the most clinically (e.g. people who are not able to achieve durable viral suppression with oral medication for reasons other than resistance to cabotegravir or rilpivirine). We operationalize these as programme outcomes to monitor the impact of our strategies by the midpoint of the ALAI UP Project. Detailed outcome definitions are in Table S3. We deliberately focused on these programme outcomes instead of setting arbitrary utilization targets because appropriate use of LAI ART depends on availability, patient preferences, funding and system‐level factors—there is no single “correct” level of uptake by which to evaluate a programme.

### 2.4 ALAI UP Program Framework

Together, the CIT and IFT made up the ALAI UP Program Framework, which we adapted from the Interactive Systems Framework for Dissemination and Implementation (ISF) 2.0 (Figure 1) [15].

**FIGURE 1:**
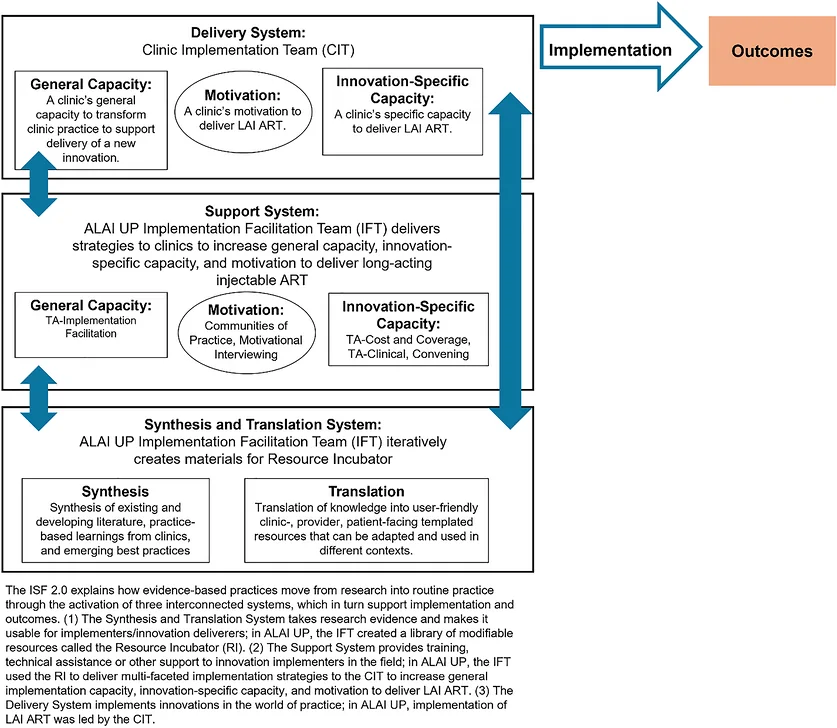
ALAI UP Program Framework adapted from the Interactive Systems Framework for Dissemination and Implementation (ISF) 2.0. ART, antiretroviral therapy; LAI, long‐acting injectable; TA, technical assistance.

### 2.5 Data Sources

To support our analysis, we used: (1) repeated measures of data submitted by clinics; (2) secondary data collected from the CIT; and (3) process data collected from the IFT. Details about all data sources are included in Table S4.

#### 2.5.1 Repeated Measures Data Submitted by Clinics

We collected four types of data from all eight clinics: (1) surveys about the clinic's iCAB/RPV programme; (2) surveys about the acceptability of ALAI UP strategies; (3) short answer narrative reports; and (4) client‐level data reports for all patients with HIV.

#### 2.5.2 Secondary Data From CIT

Clinic iCAB/RPV protocols were written by the CIT, with support from IFT (specifically the Implementation Coaching TA provider), and submitted by the project champion to ALAI UP at the end of year 1 and updated and resubmitted at the end of year 2. We reviewed these protocols as secondary data for our analyses.

#### 2.5.3 Process Data From the IFT

We drew on process data collected by the IFT, including: (1) notes from Convenings, process monitoring calls, TA calls and in‐person clinic visits; and (2) key informant interviews with the IFT.

### 2.6 Analysis

#### 2.6.1 Development of CPDs

We developed CPDs detailing a hypothesized causal pathway by which each strategy, received in eight different clinical contexts, activated mechanisms to overcome barriers, and moved clinics towards achievement of each programme outcome. CPDs are composed of strategies, mechanisms, moderators, determinants, preconditions and outcomes (see Table S5 for definitions) [16, 17]. Together, the components describe what needs to change, how changes occur, what might help or hinder change and what results are expected. We developed CPDs through an iterative four‐step process: (1) assess achievement of programme outcomes; (2) generate CPD inputs through framework analysis of ALAI UP data; (3) validate logic of CPDs with experts; and (4) conduct rapid analysis to refine CPDs. Table S6 elaborates the four‐step CPD development process.

## 3 Results

### 3.1 Programme Outcomes

Clinic progress towards programme outcomes is summarized in Table 1. A detailed narrative description of progress is included in Table S7. Of the eight clinics, one clinic fully achieved all three programme outcomes, while four clinics fully achieved two out of three outcomes and one clinic fully achieved one outcome.

**TABLE 1:** ALAI UP clinic progress towards programme outcomes at the end of year 2.

	Number of ALAI UP clinics achieving each outcome^a^		
	Fully achieved	Partially achieved	Not yet achieved
**Outcome 1**: Clinic's HIV treatment programme can provide access to information about iCAB/RPV for all patients regardless of clinical eligibility	1	5	2
**Outcome 2**: Clinic's HIV treatment programme can provide access to iCAB/RPV regardless of healthcare coverage	6	1	1
**Outcome 3**: Clinic's HIV treatment programme can provide access to iCAB/RPV for those who can clinically benefit the most.	6	0	2

^a^ Categorization of outcomes are defined in Table S3.

### 3.2 CPDs: Clinical TA Strategy for Outcome 3

In the following section, we explicate the CPD for Clinical TA Strategy (Figure 2). The CPD illustrates how and why Clinical TA operated more and less effectively to move clinics towards fully achieving Outcome 3, initiating iCAB/RPV services and prescribing iCAB/RPV to patients for whom iCAB/RPV can have the greatest clinical benefit. Case studies for how Clinical TA operated in Clinics 1 and 4 are in Figure S1a,b. CPDs for the remaining six strategies are included as Figure S2a−f and inform the discussion.

**FIGURE 2:**
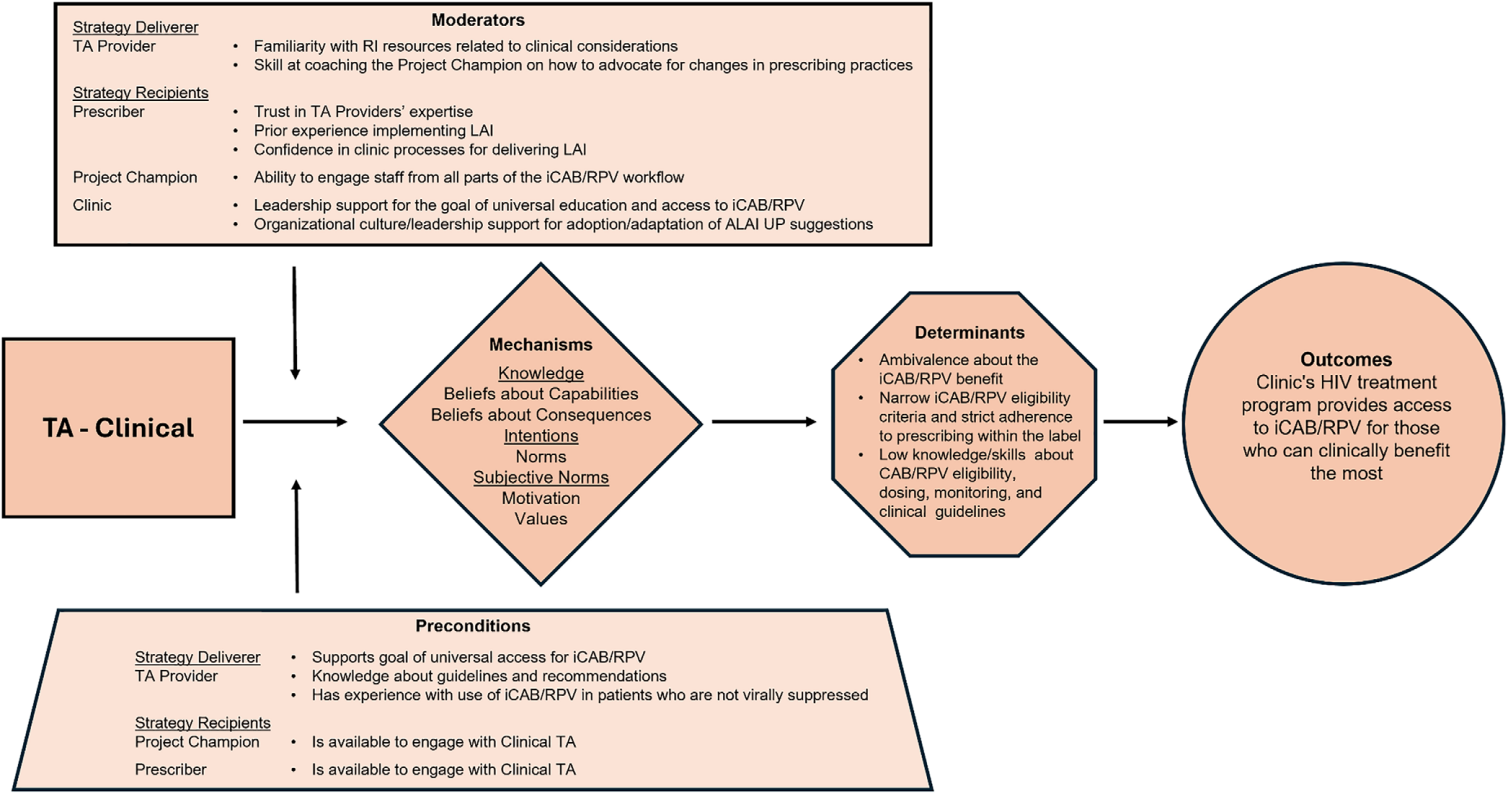
Illustrative causal pathway diagram for technical assistance‐clinical strategy. iCAB/RPV; injectable cabotegravir and rilpivirine; LAI, long‐acting injectable; RI, resource incubator. Key mechanisms are underlined.

#### 3.2.1 Determinants

Clinical TA was designed to overcome three key determinants that were barriers to clinics initiating iCAB/RPV services and expanding access to patients who could benefit the most clinically (Outcome 3). First, prescribers were ambivalent about the potential benefit of iCAB/RPV for their patients given that the iCAB/RPV label only recommended iCAB/RPV for individuals who were already durably virally suppressed. In the first year of ALAI UP, some prescribers viewed switching patients to iCAB/RPV as a “lifestyle” improvement, rather than a medication that could improve their clinical outcomes. As a result, switching to iCAB/RPV did not warrant the extra administrative labour nor risk of treatment failure or resistance when patients were already durably suppressed. Second, some prescribers took an even more conservative approach than what was detailed in the label, for example, determining that all women of childbearing age were ineligible for iCAB/RPV or requiring that patients be virally suppressed for a full year before switching to iCAB/RPV. Third, in March 2024, IAS‐USA Treatment Guidelines [18], and in September 2024, HHS Guidelines [19] were updated to recommend that iCAB/RPV be considered for people with detectable viral load when adherence difficulties prevented effective use of daily oral ART, within settings that provide extensive support services for patients. The new guidelines created a path for clinics to advocate for use of iCAB/RPV in patients with detectable viral load and for healthcare payors to cover use in such cases; in fact, under the RWHAP, clinics are required to follow the HHS guidelines. However, these recommendations took time to reach prescribers in settings outside academic medical centres, and prescribers were reluctant to implement these recommendations without more knowledge about how to dose and monitor such patients or having robust clinic infrastructure in place to support benefit navigation.

#### 3.2.2 Mechanisms

To address these determinants, the Clinical TA strategy was designed to activate mechanisms (capabilities, motivations and opportunities) to both support clinics in establishing and growing their iCAB/RPV programmes, and after the updated guidelines in 2024, offer iCAB/RPV to patients who have been unable to achieve viral suppression on oral ARTs and could be supported with wraparound services. Specifically, the strategy was hypothesized to increase *knowledge* about clinical considerations, eligibility, dosing and monitoring; increase prescriber's *beliefs in their capabilities* and the positive *consequences* of initiating a patient on iCAB/RPV; promote the *value* that having LAI ART available is a necessary part of HIV treatment services; generate *intentions* and *motivation* among prescribers to prescribe iCAB/RPV; and expose recipients to *norms* and *subjective norms* by sharing experiences learned from other clinics.

#### 3.2.3 Preconditions

In analysing which clinics fully, partially and did not yet achieve this outcome by the end of year 2, we identified preconditions for both Strategy Deliverers and Strategy Recipients that needed to be met to activate the above mechanisms. Among Strategy Deliverers, the Clinical TA provider needed to first support the goal of broad access for iCAB/RPV and believe that the benefits would outweigh the risks and burden for a clinic to offer iCAB/RPV to patients. Second, the Clinical TA provider needed to be knowledgeable about updates to clinical guidelines and recommendations. Third, the Clinical TA provider needed to have personal experience with delivery of iCAB/RPV for patients who were not virally suppressed both for credibility and to provide meaningful assistance. Among Strategy Recipients, the project champion and, in particular, the clinic prescribers had to make time and be willing to engage with Clinical TA.

#### 3.2.4 Moderators

We identified the following moderators, which facilitated or hindered whether the Clinical TA strategy activated intended mechanisms and led to achievement of Outcome 3. Among Strategy Deliverers, the Clinical TA strategy was most effective when Clinical TA providers were familiar with, and referred prescribers to, ALAI UP‐created resources to increase knowledge about prescribing and monitoring patients on iCAB/RPV, and when Clinical TA providers were skilled at coaching clinic project champions on how to advocate for expanded access to iCAB/RPV for patients who could benefit the most clinically. Among Strategy Recipients, the strategy was most successful when the project champion and prescribers trusted the clinical expertise of the TA provider, and when the project champion and prescribers had prior implementation experience with iCAB/RPV or other LAI. Additionally, the extent to which clinic prescribers supported the goal of access to iCAB/RPV for all clinically eligible patients, and specifically patients without sustained viral suppression, played a critical role in enhancing the impact of the strategy. Lastly, at clinics where prescribers and leadership were not yet on board with expanded access to iCAB/RPV, the extent to which the project champion was skilled at obtaining buy‐in from clinic prescribers to expand access, and the extent to which clinic organizational culture and leadership supported the adoption or adaptation of suggestions from ALAI UP, was very important.

## 4 Discussion

The question of how to optimize long‐acting injectable therapies in routine use is a high‐priority topic. Randomized clinical trials have established efficacy, but we still have relatively limited rigorous work in real‐world programme settings to describe how to actually implement these interventions. We found that the multifaceted strategies delivered by ALAI UP have been largely effective in building capabilities, opportunities, and motivations of CITs to deliver LAI ART services in a manner that is attentive to access considerations. Specifically, by combining written resources, in‐person didactic and discussion‐based sessions, and longitudinal implementation coaching and technical assistance, project champions and CITs had multiple opportunities to engage with the same ideas and develop protocols and services responsive to each clinic's particular challenges in achieving enhanced access to iCAB/RPV for their patients.

We summarize four key findings and recommendations for the integration of implementation science approaches into service delivery projects.

***Finding 1. Explicit specification of causal pathways enables rapid problem‐solving and identifies intervention points***. Implementation strategies are often described as discrete “packages,” yet their effectiveness depends on the specific contexts in which they are deployed and the preconditions and moderators that enable or constrain activation of mechanisms [16]. Creating CPDs helped us articulate the determinants to address and the mechanisms to activate with each strategy. When one or more clinics were not making progress, we turned to the CPDs to consider whether it was because there was a critical precondition that was absent in a specific context (e.g. at Clinic 4) or across contexts, or whether a moderator was present or absent, and in either case, whether ALAI UP could address it in any way. CPDs, therefore, provided us with a structured way to organize what we were learning and identify points where we could intervene to make a strategy maximally effective [16], informing changes to strategies in real‐time. For example, when we identified prescriber availability to engage with Clinical TA as a precondition, we made Clinical TA more accessible to prescribers by sharing direct phone numbers to Clinical TA providers, and scheduled all Clinical TA sessions of the Convening for a single day that prescribers could attend. Importantly, CPDs are just one conceptual approach for understanding mechanisms and theorizing. Other approaches like realist evaluations with their C+I+M = O configurations, mechanisms mapping and intervention mapping may also provide useful insights and may be better suited to theory‐building. Our goal was to highlight how CPDs were a helpful sensemaking tool for our project, but other projects may benefit from a less structured analytic approach.

***Finding 2. Building implementation science into service delivery projects provides opportunities for generating knowledge that can be applied broadly across clinics***. ALAI UP intentionally selected clinics that differed across multiple dimensions (location, type of agency, staffing) because we believed in the learning that can occur by studying single cases deeply [20, 21]. The small total number of clinics and their variety in context and barriers makes it challenging to draw conclusions about the effectiveness of discrete strategies or the multifaceted strategy as a whole, as one would from a randomized controlled trial. Additionally, given that ALAI UP is a service delivery and not research project, it was not feasible to measure all aspects of implementation (e.g. mechanisms). However, through our specification of strategies, detailed knowledge about how strategies operated in each clinic, and, after the end of the project in 2026, linking these programme outcomes with implementation and patient outcomes, we will have a robust roadmap and implementation tools for how to optimally support different kinds of clinics to introduce LAI ART. Additionally, we expect that these lessons will be useful to accelerate implementation of future innovations to prevent and treat HIV.

***Finding 3. Multidisciplinary implementation teams, including a clinician, are necessary to support individual champions in introducing complex biomedical interventions into practice***. Two of the most consistent moderators across all strategies were the knowledge, skills, and expertise of the project champion and the extent to which the champion was able to engage clinic staff involved in each part of the iCAB/RPV workflow in ALAI UP activities.

We knew from the outset that having a multidisciplinary implementation team to support the project champion in leading the implementation of a costly, biomedical intervention would be necessary thus, we asked clinics to identify and convene a multidisciplinary CIT [22, 23]. Implementing LAI ART was a product of the IFT working with a team (CIT) to solve problems at various levels and make clinic‐level changes. Implementing LAI ART required more than what is normally required to introduce a new oral medication: clinics needed to adapt workflows, physical space, staffing and processes to accommodate a new mode of care. Having a multidisciplinary implementation team harnesses the power, influence and expertise across multiple people who bring different capabilities and motivation [24, 25, 26]. However, we did not anticipate how difficult and unrealistic it would be for all members of the CIT to participate in all ALAI UP activities. In practice, CIT members were not exposed to all ALAI UP activities. This meant that there was more responsibility for the project champion to disseminate ALAI UP knowledge and values to their teams, and thus the effectiveness of strategies was moderated by the dissemination skills and authority of the project champion. We were not prescriptive enough to clinics about the required experience, commitment, and authority of their champion, which resulted in champions of varying degrees of effectiveness. We recommend a more explicit specification of the champion role in the future [27].

Second, participation of prescribers differed across CITs but was an important driver of the ability to move towards prescribing iCAB/RPV for eligible and interested people with HIV, and specifically for people living with HIV who are not durably virally suppressed. Notably, the two clinics in which a prescriber was not a member of the CIT did not create a protocol for viraemic patients nor initiate any viraemic patients on iCAB/RPV. Prescriber ambivalence operated as a barrier when the CIT did not successfully engage prescribers and, therefore, could not activate the necessary mechanisms to bring about a change in knowledge, values, intention or motivation. This finding is supported by previous studies which have found that inclusion of a clinician champion on implementation teams is almost always necessary for complex biomedical innovation to succeed [28, 29, 30].

***Finding 4. Responsively delivered strategies cannot overcome structural access constraints***. Organizational, local, state and national policies either constrained or enabled clinic capacity to achieve access‐related goals and outcomes. Sustainable access to LAI ART required not only successful clinical adoption and team coordination but also navigation of complex healthcare payor systems, state insurance and RWHAP ADAP policies [31, 32]. For example, while 42 states had added iCAB/RPV to their RWHAP ADAP formulary by January 2024, one in five RWHAP ADAP clients lived in a state whose RWHAP ADAP formulary did not cover iCAB/RPV [33]. In addition, RWHAP ADAP clients were not eligible for the manufacturer's patient assistance programme, which covers the cost of iCAB/RPV for patients who are uninsured, rendering iCAB/RPV inaccessible to all RWHAP ADAP clients in eight states that had not added iCAB/RPV to the RWHAP ADAP formulary.

These examples demonstrate that across the eight clinics, state policy context operated as a precondition over which clinics have no control. While written resources developed in the resource incubator, implementation coaching TA, and cost and coverage TA built CIT capabilities, and motivational interviewing and multisite convenings increased CIT motivation and created opportunities for change, the impact of ALAI UP implementation strategies was limited in the face of structural policy barriers. This finding underscores a central tension in implementation: even the most effective and responsively delivered clinic‐level implementation strategies cannot overcome structural policy barriers.

## 5 Conclusions

ALAI UP's use of CPDs as scaffolding to bring analytic clarity to the study of implementation process positions the project to develop a refined implementation strategy package to support clinics across the United States who have not yet initiated iCAB/RPV implementation. We present a case example of how to use implementation science tools in service delivery projects to inform rapid learning and adaptation throughout the project life cycle. We recommend that projects that deliver implementation strategies at the clinic level require multidisciplinary CITs that include members who can bring knowledge, skills and authority from across all phases of the workflow, as well as at least one member with lived experience. We also recommend dual project champions (one prescriber, one administrator), who have the facilitation and dissemination skills to share learnings with clinic staff and leadership. Developing CPDs that are shared with IFTs early, and adapted over the course of learning, can help build team alignment around goals and identify intervention points when strategies are not working as expected. Finally, CPDs have been useful to identify specific mechanisms, moderators and preconditions that can be prospectively measured in a future hybrid effectiveness‐implementation trial to determine which combination of strategies most effectively supports implementation of LAI ART in ways that close the gap in health outcomes.

## Author Contributions

KM and NN conceived of the ALAI UP project and designed the approach and analyses underlying the results reported here. JZ, SAG, BAK, MK, CAC, MB, MB‐L, JB, DC, CC, JE and EF contributed to the activities described as members of the implementation facilitation team. OA, KB, MC, WH, BRH, SL, CR and CS contributed by driving implementation of LAI ART at participating clinics, coordination with clinic staff, and collection of implementation and clinical data. KM, NN and CF conducted the analysis of implementation processes and outcome data and interpreted the findings in relation to the implementation frameworks guiding the study. KM and NN drafted the manuscript. MM, JZ, SAG, BAK and MK read early drafts and provided extensive feedback on the manuscript. All authors reviewed and edited the manuscript, approved the final version, and agreed to be accountable for the accuracy and integrity of the work.

## Funding

The Health Resources and Services Administration (HRSA), Department of Health and Human Services (HHS), provided financial support for this project. The award provided 100% of total costs and totalled $7,450,000.

## Conflicts of Interest

The authors declare no conflicts of interest.

## Disclaimer

The contents are those of the presenters. They may not reflect the policies of HRSA, HHS or the U.S. Government.

## Supporting information




**Table S1**: ALAI UP Clinic Context and Characteristics
**Table S2**: ALAI UP Implementation Strategy Specification
**Table S3**: ALAI UP Program Outcome Data Sources and Definitions and Assessment Criteria for ALAI UP Program Outcomes
**Table S4**: Causal Pathway Diagram Data Sources
**Table S5**: Definitions of Components included in Causal Pathway Diagrams of ALAI UP Implementation Strategies
**Table S6**: Four‐step Process for Developing and Refining Causal Pathway Diagrams
**Table S7**: Narrative Summary of ALAI UP Clinic Progress towards Program Outcomes at the End of Year 2
**Figure 1a**: Case Example illustrating Partial Achievement of Outcome 3 through Clinical Technical Assistance in Clinic 4
**Figure 1b**: Case example illustrating full achievement of Outcome 3 through Clinical Technical Assistance in Clinic 1
**Figure 2a**: Causal Pathway Diagram for ALAI UP Resource Incubator Strategy
**Figure 2b**: Causal Pathway Diagram for ALAI UP Implementation Coaching Technical Assistance Strategy
**Figure 2c**: Causal Pathway Diagram for ALAI UP Cost and Coverage Technical Assistance Strategy
**Figure 2d**: Causal Pathway Diagram for ALAI UP Motivational Interviewing Training Strategy
**Figure 2e**: Causal Pathway Diagram for ALAI UP In‐Person Multisite Convening Strategy
**Figure 2f**: Causal Pathway Diagram for ALAI UP Virtual Community of Practice Strategy

## Data Availability

The data that support the findings of this study are available from the corresponding author upon reasonable request.
