# Supplementary material for: Specification and Causal Pathway Diagramming of Implementation Strategies to Support Development of Access‐Enhancing Injectable HIV Treatment Programmes at Eight Diverse Health Clinics Across the United States
Source: J Int AIDS Soc. 2026 Oct 8;29(Suppl 5):e70190. doi: 10.1002/jia2.70190 (PMC13650423; doi:10.1002/jia2.70190)
Supplement: Supplementary file 1 [file JIA2-29-e70190-s001.docx]

**SUPPORTING INFORMATION**

**Supplemental Table 1. ALAI UP Clinic Context and Characteristics**

ALAI UP clinics were selected to be varied with respect to implementation stage (pre-implementation through implementation of LAI ART), organizational type (HIV Service Organizations, academic medical centers, safety net hospitals, community-based organizations), and geographic region across the US. Clinic mix was prioritized to generate unique but generalizable knowledge about LAI ART implementation across the country.

|  | **ALAI UP Clinics** | | | | | | | |
| --- | --- | --- | --- | --- | --- | --- | --- | --- |
|  | **1** | **2** | **3** | **4** | **5** | **6** | **7** | **8** |
| **Clinic Context** | | | | | | | | |
| Location | Dallas, Texas | San Antonio, Texas | Chicago, Illinois | Biloxi, Mississippi | Baltimore, Maryland | Orlando, Florida | Decatur, Georgia | New York City, New York |
| Region in the United States | South | South | Midwest | South | Northeast | South | South | Northeast |
| Jurisdiction Population size | 1,317,163 | 1,526,656 | 2,721,308 | 48,144 | 568,271 | 334,854 | 24,482 | 8,478,072 |
| Jurisdiction HIV prevalence per 100K population, 2023^1^ | 958 | 435 | 611 | 428 | 2,225 | 778 | 1,503 | 1,785 |
| **Clinic Characteristics** | | | | | | | | |
| Healthcare Facility Type | Community Based Organization | Community Based Organization | Safety Net Hospital | Federally Qualified Health Clinic | Department of Health Clinic | Department of Health Clinic | HIV Service Organization | Academic Medical Center Clinic |
| Total number of persons with HIV receiving care as of ALAI UP start (3/1/2023) | 133 | 371 | 451 | 600 | 691 | 2,073 | 2,134 | 3,107 |
| Total number (%) of persons with HIV ever on iCAB/RPV as of ALAI UP start (3/1/2023) | 1 (<1%) | 0 (0%) | 13 (3%) | 0 (0%) | 7 (1%) | 1 (<1%) | 23 (1%) | 11 (<1%) |

1 Data Source: Centers for Disease Control and Prevention. (2026). *NCHHSTP AtlasPlus: HIV surveillance data* [Data set]. Retrieved June 23, 2026, from [https://gis.cdc.gov/grasp/nchhstpatlas/tables.html](https://nam02.safelinks.protection.outlook.com/?url=https%3A%2F%2Fgis.cdc.gov%2Fgrasp%2Fnchhstpatlas%2Ftables.html&data=05%7C02%7Cnn2442%40cumc.columbia.edu%7Ca4dd8c36646e4e0b2d4908ded1665f9b%7Cb0002a9b0017404d97dc3d3bab09be81%7C0%7C0%7C639178434774420963%7CUnknown%7CTWFpbGZsb3d8eyJFbXB0eU1hcGkiOnRydWUsIlYiOiIwLjAuMDAwMCIsIlAiOiJXaW4zMiIsIkFOIjoiTWFpbCIsIldUIjoyfQ%3D%3D%7C0%7C%7C%7C&sdata=OJuJ2U3Nz9iavGbb%2BKb2Q0kIC73Ws4aDrXJ%2Fr2NK%2FR8%3D&reserved=0)

**Supplemental Table 2. ALAI UP Implementation Strategy Specification**

In the following specification table, we cross walked ALAI UP strategies with ERIC (Expert Recommendations for Implementing Change) strategies and specified what was delivered (action), who within the Implementation Facilitation Team (IFT) delivered the strategy (actor), who within the Clinic Implementation Team (CIT) at the clinic received the strategy (target of action - who), which mechanisms the strategies were designed to activate (target of action - mechanisms), when was it delivered (temporality within the ALAI UP Project), for how long and at what frequency (dose), and the intended outcome of the strategy [1,2]. Strategies are groups by the primary mechanism of action (capability, opportunity, motivation).

| **ALAI UP Strategy  (ERIC strategy)** | **Actor** | **Action** | **Target of Action (person)** | **Target of Action (mechanism)** [3] | **Outcome** | **Temporality within ALAI UP Project** | **Dose** |
| --- | --- | --- | --- | --- | --- | --- | --- |
| **Primary Mechanism Targeted: Capability** | | | | | | | |
| Resource Incubator (Develop educational materials; promote adaptability; distribute educational materials) | Social psychologist, Implementation scientist, and health department | Provide clinics with modifiable resources tailored to clinic implementation needs including: passive and active patient education materials; job aids to support patient education, coverage investigation, medication procurement, clinical eligibility assessments, medication administration, and patient retention; and staff training slides. | Project champion and clinic implementation team | Increase knowledge, support memory, attention and decision processes for iCAB/RPV workflow, generate norms, increase motivation to offer iCAB/RPV, communicate education and access values, create visual cues to support workflow in the environment | Clinic's HIV treatment program can sustainably provide (1) access to information about iCAB/RPV for all patients, (2) access to iCAB/RPV medication regardless of insurance status, and (3) access to iCAB/RPV for those who can clinically benefit the most. | Y1 Q2-Y2 Q4 | 36 adaptable resources |
| TA – Coverage & Cost (Provide local TA and ongoing consultation, Conduct ongoing training) | Subject matter experts (pharmacists) from academic medical centers and health department | Deliver training and as-needed support on processes that facilitate coverage of the cost of iCAB/RPV, including benefits investigation, coverage coordination and navigation, and clinic reimbursement. | Clinic staff responsible for benefits navigation, procurement, and logistics (e.g., pharmacists, case managers, benefits managers) | Increase knowledge about navigating iCAB/RPV cost and coverage, increase CIT's beliefs about their capabilities, generate optimism and beliefs about consequences from ensuring access, promote values, generate intentions, and set goal to navigate access regardless of payor, and share norms across clinics. | Clinic's HIV treatment program can sustainably provide access to iCAB/RPV medication regardless of insurance status | Y1Q2 – Y2B2 | Navigating cost and coverage trainings during Convenings (3); Ad-hoc, by request TA virtually and during site visits |
| TA-Clinical (Provide local TA and ongoing consultation, Conduct ongoing training)) | Subject matter experts - clinicians from academic medical centers and health department | Deliver clinical training using a mix of didactic and case-based methods for clinic staff on iCAB/RPV use, including eligibility assessment, initiation, maintenance, and discontinuation protocols. | Project champion and prescribers | Increase knowledge about clinical considerations, eligibility, dosing and monitoring, increase prescriber’s beliefs in their capabilities and the positive consequences of initiating a patient on iCAB/RPV, promote value that having LAI ART available is a necessary part of HIV treatment services; generate intentions and motivate prescriber to prescribe iCAB/RPV, demonstrate norms and subjective norms by sharing experiences across clinics. | Clinic's HIV treatment program can sustainably provide (1) access to information about iCAB/RPV for all patients, and (3) access to iCAB/RPV for those who can clinically benefit the most. | Y1Q2 - Y2B2 | Clinical trainings during Convenings (3); Ad-hoc/by-request clinical trainings virtually and during in person clinic visits |
| **Primary Mechanism Targeted: Opportunity** | | | | | | | |
| In-Person Multisite Convenings (Conduct implementation team meetings; Conduct educational meetings; capture and share local knowledge) | Implementation scientists, clinical educators, and TA providers | Hold in-person collaborative meeting to share best practices across clinics, build within clinic team cohesion, provide time to work on protocols, enhance clinical and non-clinical knowledge, and prepare for sustainability and scale-up of LAI ART. | Project champion and at least two other members of clinic implementation team | Increase knowledge about all aspects of the iCAB/RPV workflow, increase beliefs about capabilities and optimism through peer-to-peer sharing, build intention and motivation to build an access-enhancing LAI ART program, leverage social influences and environmental context and resources to build norms that LAI ART are a critical component of HIV treatment. | Clinic's HIV treatment program can sustainably provide (1) access to information about iCAB/RPV for all patients, (2) access to iCAB/RPV medication regardless of insurance status, and (3) access to iCAB/RPV for those who can clinically benefit the most. | Y1 Q2; Y1, Q3; Y2,Q3 | Three 2.5-day in person meetings |
| **Primary Mechanism Targeted: Motivation** | | | | | | | |
| TA-Implementation Coaching (Implementation Facilitation) | TA providers from academic medical institutions, AIDS Education and Training Center, and health department | Provide ongoing implementation support in response to clinic-identified needs; facilitate connections with subject matter experts to overcome identified barriers; support development of SMARTIE goals and track progress toward goals. | Project champion and clinic implementation team | Increase knowledge about iCAB/RPV implementation, increase CIT's beliefs about their capabilities, generate optimism and belief in the positive consequences from implementing iCAB/RPV, promote value, generate intentions, and set goal to develop an access-enhancing program; share norms across clinics. | Clinic's HIV treatment program can sustainably provide (1) access to information about iCAB/RPV for all patients, (2) access to iCAB/RPV medication regardless of insurance status, and (3) access to iCAB/RPV for those who can clinically benefit the most. | Y1Q2-Y2Q4 | 9 monthly TA calls in YR1;  6 bi-monthly TA calls in YR2; additional TA calls as requested by clinic; additional topic-specific training as requested by clinic |
| Motivational Interviewing Training (Conduct ongoing training) | Motivational interviewing trainer | Provide training and ongoing coaching to improve direct service staff’s understanding of MI concepts and to strengthen their capacity to implement MI techniques in their practice to better engage and retain LAI ART patients. | Direct service staff with patient contact | Increase knowledge and skills related to recognizing change talk in clients and colleagues, increase the intention and motivation to use MI techniques in patient-centered communications, increased recognition of the value of educating all patients about HIV treatment options | Clinic's HIV treatment program can sustainably provide (1) access to information about iCAB/RPV for all patients, (2) access to iCAB/RPV medication regardless of insurance status, and (3) access to iCAB/RPV for those who can clinically benefit the most. | Y1Q2, Y1Q3, Y2Q1-Q2 | One 1-hour session at Convening 1; One ½ day session at Convening 2; 12 monthly MI coaching sessions; One ½ day in person training at two clinics |
| Virtual Community of Practice (Create a learning collaborative) | AIDS Education and Training Center | Organize and facilitate virtual sessions for cross-clinic exchange to share challenges and lessons learned on specific topics between in person convenings. | Clinic implementation teams | Increase knowledge about all aspects of the iCAB/RPV workflow, increase beliefs about capabilities and optimism through peer-to-peer sharing, build intention and motivation to build an access-enhancing LAI ART program, leverage social influences to build norms that LAI ART are a critical component of HIV treatment | Clinic's HIV treatment program can sustainably provide (1) access to information about iCAB/RPV for all patients, (2) access to iCAB/RPV medication regardless of insurance status, and (3) access to iCAB/RPV for those who can clinically benefit the most. | Y1Q2-Y2Q3 | 7 virtual bi-monthly CoP meetings |

CIT: Clinic Implementation Team

ERIC strategies: Expert Recommendations for Implementing Change strategies

iCAB/RPV: Injectable cabotegravir/rilpivirine

LAI ART: Long-acting injectable antiretroviral therapy

MI: Motivational Interviewing

TA: Technical Assistance

**Supplemental Table 3. ALAI UP Program Outcome Data Sources and Definitions and Assessment Criteria for ALAI UP Program Outcomes**

| **Outcomes** | **Data Source** | **Definition** |
| --- | --- | --- |
| **Outcome 1:** Clinic's HIV treatment program can provide access to information about iCAB/RPV for all patients regardless of clinical eligibility | To assess Outcome 1, we reviewed clinics’ iCAB/RPV protocols; IFT notes from process monitoring calls, TA calls, clinic visits, and Convenings; and client-level data from each clinic. | **Fully achieved:** Clinics fully achieved the first outcome if they met at least one of the following conditions: the clinic has had policies in place to actively and/or passively educate all patients with HIV about iCAB/RPV regardless of the patients’ clinical eligibility (as documented in their iCAB/RPV protocol) and implemented these policies with fidelity for >12 months (as documented in process monitoring, TA, clinic visit, or Convening notes; and/or the clinic has documented educating ≥98% of their patients with HIV about iCAB/RPV.  **Partially achieved:** Clinics partially achieved this outcome if they demonstrated an intention in their protocol to actively and/or passively educate all patients with HIV about iCAB/RPV, but this protocol has been in place for <12 months and/or the clinic has not yet documented education of ≥98% of their patients with HIV about iCAB/RPV.  **Not yet achieved:** Clinics have not yet achieved this outcome if they have not yet met any of the above conditions. |
| **Outcome 2:** Clinic's HIV treatment program can provide access to iCAB/RPV medication regardless of insurance coverage | To assess Outcome 2, we reviewed the clinic’ surveys about their iCAB/RPV programs, TA call notes, and clinic visit notes. | **Fully achieved:** Clinics fully achieved the second outcome if they were able to provide iCAB/RPV to patients at their clinic across all payor types (including uninsured patients) by the end of year 2.  **Partially achieved:** Clinics partially achieved the second outcome if they were able to provide iCAB/RPV for only some payor types or replied “it depends” for some payor types, but they were taking active steps (as documented in their protocol and TA call and clinic visit notes) to increase access for all patients (e.g., by procuring insurance for uninsured patients or advising patients to switch insurance plans to one that covered iCAB/RPV).  **Not yet achieved:** Clinics have not yet achieved this outcome if they have not yet met any of the above conditions. |
| **Outcome 3:** Clinic's HIV treatment program can provide access to iCAB/RPV for those can clinically benefit the most (e.g. have not been able to achieve durable viral suppression with oral therapy for reasons other than resistance to rilpivirine) | To assess Outcome 3, we reviewed the clinics’ iCAB/RPV protocol and client-level data. | **Fully achieved:** Clinics fully achieved this outcome if they have grown their iCAB/RPV program since starting ALAI UP and (1) have helped >1 patient with viral load>200 copies/mL achieve viral suppression while on oral ART explicitly to switch to iCAB/RPV, and/or (2)  have initiated >1 patient on iCAB/RPV with a pre-iCAB/RPV viral load of >200 copies/mL or a history of viral non-suppression.  **Partially achieved:** Clinics partially achieved this outcome if they have grown their iCAB/RPV program and demonstrated an intention to (1) help a patient with viral load>200 copies/mL achieve viral suppression while on oral ART explicitly to switch to iCAB/RPV, and/or (2) initiate a patient on iCAB/RPV with a VL>200 copies/mL or history of viral non-suppression as evidenced in their iCAB/RPV protocol, but have not yet done (1) or (2).  **Not yet achieved:** Clinics have not yet achieved this outcome if they have not yet met any of the above conditions. |

iCAB/RPV: Injectable cabotegravir/rilpivirine

IFT: ALAI UP Implementation Facilitation Team

TA: Technical Assistance

VL: Viral load

**Supplemental Table 4. Causal Pathway Diagram Data Sources**

| **Data Type** | **Frequency of data collection** | **Data collection method** | **Description** |
| --- | --- | --- | --- |
| ***Repeated Measures Data Collected from Partner Sites*.** | | | |
| Surveys about the clinics’ iCAB/RPV programs | Year 1: Quarterly  Year 2: Bi-annually | Administered via REDCap and completed by the project champion | Surveys about the clinics’ iCAB/RPV program included questions about clinics’ overall staffing for HIV care and iCAB/RPV care, number of clients on oral ART and iCAB/RPV served by the clinic in a given period, availability of wrap-around services to support iCAB/RPV access and retention, clinic-level strategies for educating patients about iCAB/RPV, PWH’s insurance mix, and access to iCAB/RPV by insurance type. |
| Surveys about the acceptability of ALAI UP strategies | Year 1: Quarterly  Year 2: Bi-annually | Administered via REDCap and completed by the project champion | Surveys about the acceptability of ALAI UP strategies assessed the following four domains of the Theory of Acceptability [4]: perceived effectiveness, affective attitude, burden, and opportunity cost. |
| Surveys about the acceptability of Convening sessions | Years 1 and 2: Annually | Paper surveys completed by Convening attendees | All attendees of the In-Person Multisite Convening were asked to complete acceptability surveys rating the perceived effectiveness of each session of the Convening immediately after the meeting. |
| Short answer narrative reports | Year 1: Quarterly  Year 2: Bi-annually | Administered via REDCap and completed by the project champion | The narrative reports included open-ended short answer questions about the clinics’ successes, challenges, and changes related to iCAB/RPV implementation. Specifically, project champions were asked to report accomplishments and challenges related to iCAB/RPV implementation, steps the clinic has taken to ensure access to iCAB/RPV, any changes in the internal and external context that have impacted iCAB/RPV implementation, unintended consequences related to iCAB/RPV implementation, and impact stories. |
| Client-level data report for all patients with HIV | Year 1: Quarterly  Year 2: Bi-annually | Excel file prepared by the clinic’s data manager/expert in collaboration with the project champion or other members of the CIT and uploaded onto REDCap | The client-level data report included data for all patients with HIV, specifically data on iCAB/RPV clinical eligibility, iCAB/RPV education status, iCAB/RPV payor coverage status, and iCAB/RPV initiation status for all patients with HIV. |
| ***Secondary Data Collected from Clinic Implementation Team*.** | | | |
| Clinic iCAB/RPV protocols | Submitted at the end of year 1 and then revised/updated and resubmitted at the end of year 2 | Word document written by CIT | Clinic iCAB/RPV protocols were written by the CIT, with support from IFT (specifically the TA provider). Development of a clinic’s specific iCAB/RPV protocol was a program requirement of ALAI UP. Each clinic’s protocol detailed the clinics’ specific processes for iCAB/RPV implementation, including engaging patients; navigating coverage and cost; procurement and storage' prescribing and administering; and supporting retention. |
| ***Process Data from the Implementation Facilitation Team*.** | | | |
| Implementation notes from Convenings | Years 1 and 2: Annually | Word document | Convenings notes were captured by members of the IFT in a Word document. |
| Implementation notes from process monitoring calls | Year 1: Monthly | Word document | Process Monitoring calls were conducted by the IFT process monitoring team with the CIT and notes were captured in an unstructured Word document. |
| Implementation notes from TA calls | Year 1: Monthly  Year 2: Monthly | Word document | TA calls were conducted by the TA provider with the CIT and notes on clinic implementation progress, barriers and facilitators, TA needs, and engagement with ALAI UP strategies were captured in a structured debrief Word document form by the TA provider. |
| Implementation notes from in-person clinic visits | End of year 2 | Word document | In-Person Clinic Visits were conducted by the TA provider and members of the IFT to observe the nature and quality of iCAB-RPV implementation and fidelity to written protocol and notes were captured in a structured clinic visit report. |
| Key informant interviews with the IFT transcripts | Middle of Year 3 | Word document | Key informant interviews with implementation strategy creators and/or deliverers, and TA providers were conducted in year 3 to refine the causal pathway diagrams. Interviews were conducted virtually over Microsoft Teams and recorded and transcribed. |

CIT: Clinic Implementation Team

iCAB/RPV: Injectable cabotegravir/rilpivirine

IFT: ALAI UP Implementation Facilitation Team

TA: Technical Assistance

REDCap: Research Electronic Data Capture

**Supplemental Table 5. Definitions of Components included in Causal Pathway Diagrams of ALAI UP Implementation Strategies**

Causal Pathway Diagrams (CPDs) are graphical representations that visualize the causal processes through which implementation strategies work to achieve intended outcomes [5]. These box and arrow diagrams can be used to generate hypotheses of cause-and-effect relationships and mechanisms that connect strategies with the outcomes they are designed to impact as well as preconditions and moderators of these casual relationships [6]. Causal pathway diagrams are comprised of determinants, moderators, preconditions, mechanisms and outcomes. Together, the components describe what needs to change, how it changes, what might help or hinder that change, and what results are expected.

We describe and report on the use of CPDs to achieve three objectives: (1) define the goals of each ALAI UP implementation strategy and the theoretical processes by which the goals can be achieved; (2) identify preconditions and moderators that influence the strategies’ impact on program goals; and (3) demonstrate how this process can inform the iteration and refinement of strategies to be tested and evaluated in future research studies. The audience for this manuscript includes funding agencies, evaluation and technical assistance providers, capacity building and training centers, implementation scientists, and program administrators and care providers wishing to leverage best practices to integrate LAI ART into HIV care.

| **Components** | **Definitions** |
| --- | --- |
| Determinants | Determinants are context-specific factors that influence implementation, functioning as barriers or facilitators to achieving desired outcomes. In CPDs, the focal determinant is the specific barrier or facilitator that the strategy intends to address in order to change downstream outcomes. In the CPDs developed for ALAI UP strategies, the determinants were similar across all clinics. |
| Moderators | Moderators are factors that strengthen or weaken the relationship between two elements in the causal pathway, such as between a strategy and a mechanism or between a mechanism and an outcome. They can either strengthen or weaken the impact of a strategy and may operate at any point along the causal chain. In the CPDs presented here we focus on moderators between the strategy and the mechanism. |
| Preconditions | Preconditions are necessary factors that must be in place for part of the causal process to occur, such as for a mechanism to be activated or for a determinant to be affected. If a precondition is not met, the strategy cannot exert its intended effect along that part of the pathway, effectively blocking that segment of the causal chain. In the CPDs presented here we focus on preconditions between the strategy and the mechanism. |
| Mechanisms | Mechanisms are the processes, events, or responses through which an implementation strategy produces change in the targeted determinant and, ultimately, outcomes. They capture the “how” or “why” the strategy works (e.g., increasing knowledge or skills, building norms or activating social influences) and should be articulated so they can be observed, measured, or inferred.  In the CPDs presented here we draw from 26 mechanisms of actions set out by Connell et al [3]. |
| Outcomes | Outcomes in CPDs typically include proximal outcomes (early, observable indicators that a mechanism is activated or a determinant is changing) and distal implementation outcomes (such as adoption, fidelity, penetration, or sustainability). Proximal outcomes serve as early signals that the strategy is operating as intended, while distal outcomes represent the longer-term implementation changes the strategy ultimately aims to achieve. In ALAI UP CPDs we use the term “program outcomes” to describe the key features of an LAI ART program that will enable it to lead to distal outcomes, namely decreasing disparities in HIV outcomes. |

**Supplemental Table 6. Four-step Process for Developing and Refining Causal Pathway Diagrams**

| **Step 1: Assess Achievement of Program Outcomes** | We assessed each clinic’s achievement of the three program outcomes by reviewing the clinics’ iCAB/RPV protocols; clinic survey responses about their iCAB/RPV programs; IFT notes from process monitoring calls, TA calls, clinic visits, and convenings; and client-level data from each clinic. Clinic achievement was categorized as “fully,” “partially,” or “not yet achieved” for each of the three outcomes (see Supporting Information Table 4 for outcome definitions). |
| --- | --- |
| **Step 2. Generate CPD Inputs through Framework Analysis** | To develop a preliminary causal pathway diagram (CPD) for each strategy that could explain why clinics fully, partially, or had not yet achieved the program outcomes, we used framework analysis to review all sources of data related to a specific strategy (familiarization) and coded the data as determinant, moderator, precondition, or mechanism (indexing), resulting in a matrix with CPD components in each column and strategies in each row [7,8]. The preliminary CPDs that resulted were intended to be exhaustive and encompass experiences across all clinics. |
| **Step 3. Validate the Logic of CPDs with Experts** | To analyze and refine the preliminary CPDs, we enlisted the experience and expertise of strategy creators and deliverers through 13 semi-structured interviews. “Strategy creators and deliverers” refer to individuals involved in the development and/or delivery of the implementation strategies, including those responsible for designing, adapting, or operationalizing strategies The goal of these interviews was to test our draft CPDs for each strategy to the experience of each clinic (with each clinic serving as a case study) to generate a global CPD that could explain the causal pathways for each strategy across all clinics. At each interview, we presented the relevant preliminary CPD and talked through each component of the CPD, inviting the creator/deliverer to ask clarifying questions about the content and suggest changes based on their experience, intentions in creating/delivering the strategy, and expertise. For strategy deliverers who interacted directly with clinics as TA providers, we asked interviewees to talk aloud about how each of the strategies operated in practice at each clinic they supported. |
| **Step 4. Conduct Rapid Analysis to Refine CPDs** | After completing all interviews, we used rapid analysis to refine the CPDs to incorporate insights from strategy creators and deliverers [9]. This led to the creation of a final, global CPD for each strategy that included all possible determinants, moderators, preconditions, and mechanisms identified across the eight clinics. These final CPDs are included in the manuscript: one is explicated in the text below as an illustrative example, and the remaining six are included in Supplemental Figure 2a-f. |

CPD: Causal Pathway Diagram

IFT: Implementation Facilitation Team

**Table 7. Narrative Summary of ALAI UP Clinic Progress towards Program Outcomes at the End of Year 2**

***Outcome 1: Access to information about iCAB/RPV to all patients regardless of clinical eligibility.*** Prior to participation in ALAI UP, clinics were not educating all patients about iCAB/RPV. By the end of year 2, one clinic had fully achieved this outcome, with documented iCAB/RPV education for >98% of their patients. Five clinics had partially achieved this outcome by demonstrating an intention to educate all people with HIV about iCAB/RPV, as evidenced by a written protocol and documented education interactions. Of these five clinics, four clinics chose one-on-one active education, while one clinic chose a passive education approach (information about iCAB/RPV visible in every exam room); we categorized these clinics as partially achieving the outcome because these policies had been in place <12 months and the clinic had not yet reached all patients with iCAB/RPV education. Two clinics had not made any progress towards this outcome: one clinic did not endorse educating all patients and one had just developed iCAB/RPV workflows.

***Outcome 2: Access to iCAB/RPV medication to all patients regardless of healthcare coverage.*** Prior to participation in ALAI UP, no clinic was able to provide iCAB/RPV to all patients regardless of insurance coverage or payor type. By the end of year 2, six clinics fully achieved this outcome. One clinic partially achieved this outcome by providing access to iCAB/RPV to all but uninsured patients and by the end of year two had invested resources to provide insurance for uninsured patients so that they could access iCAB/RPV; however, about a quarter of their patients remained uninsured and therefore did not have access. One clinic had not yet achieved this outcome. It is located in a state where iCAB/RPV was not on the Ryan White HIV/AIDS Program (RWHAP) AIDS Drug Assistance Program (ADAP) formulary [10] and had not yet identified workarounds to provide iCAB/RPV to the large proportion of patients whose medications are covered through RWHAP ADAP, therefore no patients on ADAP have been initiated. However, by the end of year 2, this clinic had initiated two people with HIV with commercial insurance on iCAB/RPV.

Importantly, among the clinics who achieved this outcome, not all patients who were eligible and interested in switching to iCAB/RPV were able to do so. All clinics had a waiting list or a ceiling on the number of patients they could initiate. Scale up was intentionally limited to balance continuity of care (e.g., uninterrupted coverage and wraparound supports to remain on therapy) with each clinic’s financial and staffing constraints.

***Outcome 3: Access to iCAB/RPV for patients who can clinically benefit the most*** (e.g., people who are not able to achieve durable viral suppression with oral medication for reasons other than resistance to cabotegravir and/or rilpivirine). Before participation in ALAI UP, no clinics had helped patients achieve viral suppression for the explicit purpose of switching them to iCAB/RPV nor initiated iCAB/RPV in patients with viral loads >200 copies/mL. By the end of year 2, six clinics had fully achieved this outcome: two clinics initiated patients on iCAB/RPV with a pre-iCAB/RPV viral load >1000 copies/mL, and another two clinics initiated patients with viral loads between 200-1000 copies/mL or with a documented history of viral non-suppression. The remaining two clinics have not yet achieved this outcome.

**Figure 1a. Case Example illustrating Partial Achievement of Outcome 3 through Clinical Technical Assistance in Clinic 4**

Clinic 4 had a very active and engaged CIT and project champion who supported the goal of expanding access to iCAB/RPV, including patients with viremia. However, this clinic only had one HIV clinician who prescribed ART and this prescriber was apprehensive about iCAB/RPV, prescribing it only to patients who had been virally suppressed for >12 months (*determinants*). This prescriber was also not a part of the CIT *(moderator*) and was unwilling to make time to engage in any Clinical TA or ALAI UP activities, despite multiple attempts to engage virtually and in person (*precondition*). Therefore, it was not possible to deliver Clinical TA to this prescriber and in year 1, Clinical TA was unable to fully activate any mechanisms of this strategy. Recognizing this limitation through interaction with their dedicated TA provider, the project champion advocated for a new nurse practitioner (NP) to join the clinic’s HIV team in year 2. Having an additional prescriber at Clinic 4 was a key difference maker. The NP was willing to make time to engage with Clinical TA (*precondition*), which led to activation of multiple mechanisms: the NP attended the In-Person Multisite Meeting where the NP increased *knowledge* about iCAB/RPV through didactic sessions, strengthened *intention* to advocate for patients who could benefit from iCAB/RPV, and was exposed to the *norms* of prescribers at other ALAI UP clinics through case discussions and *subjective norms* of prescribers at other ALAI UP clinics who were initiating patients with viremia on iCAB/RPV. The NP was also exposed to the *values* of ALAI UP and *motivated* when seeing written testimonials and hearing other clinicians discuss the benefits of iCAB/RPV. This empowered the NP to join the project champion to advocate for initiating more virally suppressed patients on iCAB/RPV. Having a second prescriber at the clinic also gave the initial prescriber a peer who could share *knowledge* about new clinical guidelines gained through participation in ALAI UP and expose the initial provider to new *subjective norms* about which patients could be initiated on iCAB/RPV. After the introduction of the second provider and engagement with clinical TA, the clinic began to initiate patients on iCAB/RPV at a steady rate of ~5 clients/month and by the end of year 2 had plans to begin initiating clients with viremia who have been unable to suppress on oral ARTs.

**Figure 1b. Case example illustrating full achievement of Outcome 3 through Clinical Technical Assistance in Clinic 1**

Like Clinic 4, Clinic 1 had a very active and engaged CIT and project champion who was highly effective and supported the goal of expanding access for iCAB/RPV, including to patients with viremia. Also, like Clinic 1, Clinic 4 only had a single prescriber who was also not engaged in the CIT. However, unlike Clinic 4, the prescriber at Clinic 1 was willing to engage with Clinical TA (*precondition*). Specifically, an important encounter with a Clinical TA provider and the prescriber during an In-Person Clinic Visit was a turning point for Clinic 1. During this meeting, a Clinical TA provider gained the trust of the clinic provider (*moderator*) and presented data supporting the use of iCAB/RPV in patients with viremia (*knowledge*). This led to a clinical discussion about individual patient cases where iCAB/RPV could be considered (*subjective norms*) and prompted a broader discussion with the CIT and the prescriber about how iCAB/RPV could be used as a tool to “meet patients where they are”, which tapped into a core patient-centered *value* of the clinic. The discussion increased *motivation* and activated *intentions*, which was evidenced by the fact that the clinic initiated their first patient on iCAB/RPV with viremia shortly after the clinic visit.

CIT: Clinic Implementation Team

CPD: causal pathway diagram

NP: nurse practitioner

TA: Technical Assistance

iCAB/RPV: injectable cabotegravir/rilpivirine

**Figure 2a. Causal Pathway Diagram for ALAI UP Resource Incubator Strategy**

**Figure 2b. Causal Pathway Diagram for ALAI UP Implementation Coaching Technical Assistance Strategy**

**Figure 2c. Causal Pathway Diagram for ALAI UP Cost and Coverage Technical Assistance Strategy**

**Figure 2d. Causal Pathway Diagram for ALAI UP Motivational Interviewing Training Strategy**

**Figure 2e. Causal Pathway Diagram for ALAI UP In-Person Multisite Convening Strategy**

**Figure 2f. Causal Pathway Diagram for ALAI UP Virtual Community of Practice Strategy**

**References**

1.	Powell BJ, McMillen JC, Proctor EK, Carpenter CR, Griffey RT, Bunger AC, et al. A Compilation of Strategies for Implementing Clinical Innovations in Health and Mental Health. Med Care Res Rev MCRR. 2012 Apr;69(2):123–57.

2.	Proctor EK, Powell BJ, McMillen JC. Implementation strategies: recommendations for specifying and reporting. Implement Sci. 2013 Dec;8(1):139.

3.	Carey RN, Connell LE, Johnston M, Rothman AJ, De Bruin M, Kelly MP, et al. Behavior Change Techniques and Their Mechanisms of Action: A Synthesis of Links Described in Published Intervention Literature. Ann Behav Med [Internet]. 2018 Oct 10 [cited 2025 Nov 7]; Available from: https://academic.oup.com/abm/advance-article/doi/10.1093/abm/kay078/5126198

4.	Sekhon M, Cartwright M, Francis JJ. Acceptability of healthcare interventions: an overview of reviews and development of a theoretical framework. BMC Health Serv Res. 2017 Dec;17(1):88.

5.	Meza RD, Weiner BJ, Lewis CC, Pullmann MD, Klasnja P. Causal Pathway Diagrams: A Toolkit for Selecting, Tailoring, and Optimizing Implementation Strategies [Internet]. 2023 [cited 2025 Nov 7]. Available from: https://impscimethods.org/application/files/9817/0448/3988/ImpSci_Methods-Toolkit_Causal-Pathway-Diagrams.pdf

6.	Klasnja P, Meza RD, Pullmann MD, Mettert KD, Hawkes R, Palazzo L, et al. Getting cozy with causality: Advances to the causal pathway diagramming method to enhance implementation precision. Implement Res Pract. 2024 Jan;5:26334895241248851.

7.	Ritchie J, Spencer L. Qualitative data analysis for applied policy research. In: Bryman A, Burgess RG, editors. Analyzing qualitative data [Internet]. Abingdon, UK: Taylor & Francis; 1994 [cited 2025 Nov 11]. p. 173–94. Available from: https://www.taylorfrancis.com/books/9780203413081

8.	Spencer L, Ritchie J, Lewis J, Dillon L. Quality in Qualitative Evaluation: A framework for assessing research evidence. Government Chief Social Researcher’s Office, Cabinet Office; 2003.

9.	Lewinski AA, Crowley MJ, Miller C, Bosworth HB, Jackson GL, Steinhauser K, et al. Applied Rapid Qualitative Analysis to Develop a Contextually Appropriate Intervention and Increase the Likelihood of Uptake. Med Care. 2021 Jun;59(Suppl 3):S242–51.

10.	Zalla LC, Horn T, Lujintanon S, Lesko CR. State-level variation in access to long-acting injectable antiretroviral therapy for HIV in the United States. Health Aff Sch. 2025 Jan 29;3(2):qxaf016.
